# Gearing Time Toward Musical Creativity: Conceptual Integration and Material Anchoring in Xenakis’ *Psappha*

**DOI:** 10.3389/fpsyg.2020.611316

**Published:** 2021-01-18

**Authors:** José L. Besada, Anne-Sylvie Barthel-Calvet, Cristóbal Pagán Cánovas

**Affiliations:** ^1^ Department of Musicology, Universidad Complutense de Madrid, Madrid, Spain; ^2^ Department of Music and Musicology, Université de Lorraine, Metz, France; ^3^ Labex GREAM, Université de Strasbourg, Strasbourg, France; ^4^ Department of English Philology, Universidad de Murcia, Murcia, Spain; ^5^ Department of Quantitative Linguistics, Universität Tübingen, Tübingen, Germany

**Keywords:** conceptual integration, material anchors, time conceptualization, compositional creativity, enaction, science-based composition, Iannis Xenakis

## Abstract

Understanding compositional practices is a major goal of musicology and music theory. Compositional practices have been traditionally viewed as disembodied and idiosyncratic. This view makes it hard to integrate musical creativity into our understanding of the general cognitive processes underlying meaning construction. To overcome this unnecessary isolation of musical composition from cognitive science, in this conceptual analysis, we approach compositional processes with the analytic tools of blending theory, material anchoring, and enaction. Our case study is Iannis Xenakis’ use of sieves for distributing rhythmic patterns in *Psappha*. Though disregarded in previous accounts, the timeline and the gearwheel provide crucial conceptual templates for anchoring Xenakis’ idea of time for this score. This case study of conceptual integration templates for temporal representation seeks to gain insight into musical creativity, embodiment, and blending, especially into how virtual interactions with material structures facilitate the construction of complex meanings.

## Introduction

Compositional practices lie at the heart of the inquiry into musical creativity. Understanding the thought processes that drive composers’ inventions is one of the major goals of the musicological enterprise and also one of the most difficult to achieve. Musicology was born as a scholarly discipline in the German-speaking world of the nineteenth century, therefore echoing the romantic myth of the lone genius (Montuori and Purser, 1995), a spiritual mediator – typically a European male – between the Muses and the audience. The discipline has since then undergone considerable evolution. Now composition is increasingly regarded as a performative situation within a social milieu, and therefore as one of the multiple facets of musicking (Small, 1998). However, such an approach faces a great challenge when it comes to accessing adequate and sufficient data. While, in a concert, we may observe the behavior of musicians and audience, access to factual compositional practices is generally much more restricted.

Beyond several controlled observations and ethnographic accounts (e.g., Collins, 2005; Donin and Theureau, 2006; Donin and Féron, 2012; Clarke et al., 2013; Donin, 2017; Besada, 2018), composition is still typically studied without making explicit connections with the cognitive processes that make it possible. As a result, composers’ creative processes are largely viewed as disembodied and idiosyncratic, and thus considered separately from other cases of creativity, communication, or performance. This makes it very hard to integrate musical creativity into our understanding of the general cognitive processes at work in meaning construction. By viewing musical composition as detached from the mental capacities that underlie all other products of human thought, we are also making musical creativity unnecessarily hard to study.

Here, we argue for an approach that allows us to integrate compositional practices into a general framework for the study of human cognition. Recently, a number of studies have been struggling to place *situated cognition* at the center of our understanding of compositional creativity (e.g., Nagy, 2017; Zembylas and Niederauer, 2018; Schiavio et al., 2020). The terms *situated*, *distributed*, or *grounded cognition*, associated to *4E* (embodied, extended, embedded, and enactive) *cognition*, refer to theoretical positions holding that mental life is inherently tied to its bodily, perceptual, and sociocultural dimensions. Our study seeks not only to expose the situated nature of a seemingly disembodied compositional practice, but also – and perhaps more importantly – to analyze how the cognitive operation of advanced conceptual integration takes this situated-distributed-grounded cognition to a higher level, giving rise to both everyday and sophisticated artistic creativity.

For that purpose, our conceptual analysis will observe Iannis Xenakis’ compositional processes for *Psappha* (1975). We will analyze some visual representations and mathematical conceptions, which played a crucial role in the development of the ideas about time that he applied to this piece. This case study is particularly enlightening, because the score was written for an undetermined set of unpitched percussion instruments and with a very restricted use of rhythmic figures. It is therefore easier to just focus on a very reduced list of compositional features, thus exposing the fundamentals of the cognitive processes at work.

## Blending, Anchoring, and Time

Multiple terms refer to the unrivaled human capacity for integrating disparate experiences and knowledge structures into novel conceptual wholes: *bisociation* (Koestler, 1964), *cognitive fluidity* (Mithen, 1999), or *combinational creativity* (Boden, 2009). The most detailed theoretical framework for this cognitive ability, including its constitutive and governing principles and its patterns across human activities, is known as *blending theory* (Fauconnier and Turner, 2002). Blending theory hypothesizes that conceptual integration proceeds through dynamic mappings across *mental spaces* (Fauconnier, 1985, 1997), i.e., small conceptual packages activated for thought or action.

For example, in the presence of *inanimate secondary visual cues*, such as a carcass or a series of footprints, human beings are capable of activating a mental space with a scene in which a predator is eating the carcass or moving along a trajectory. In an *ad hoc*, adaptive process, humans can map the imagined or remembered scene onto the perceived visual stimulus, establishing correspondences – for instance, connecting the remembered paws of the predator to the perceived shape of the footprints. Crucially, human beings can also form a network that selectively projects elements from the activated mental spaces – in this case, the spaces of the imagined predator and the visible footprints – onto a blended space, where they can be recombined. This recombination leads to the emergence of previously unavailable meanings: the imagined predator now becomes a real one, absent but perhaps hiding in the surroundings, and the marks on the ground now become a *trail* leading toward, where the predator could be (Pagán Cánovas and Turner, 2016).

“Seeing” a fictive or absent reality is easy for humans, but extremely hard for any other species. Some evolutionary advanced primates can produce distinct vocalizations when perceiving different predators (Cheney and Seyfarth, 1988) but cannot generally make sense of inanimate secondary visual cues. Presumably, this imaginative skill was an evolutionary advantage first, and then turned into the basis of all human behavior, which relies on inhabiting realities that cannot be directly perceived, such as cultural conventions, institutions, nations, identities, narratives, and so forth (Fauconnier and Turner, 2002).

Conceptual blending theory hypothesizes that an advanced conceptual integration must indeed start from situated or distributed cognitive processes, but also that it drives those purposes according to its own goals and principles. An aspect of particular relevance for connecting distributed cognition processes with dynamic conceptual integration is the *material anchoring* of conceptual blends (Hutchins, 2005), by means of which the conceptual relations established through an integration network can become materialized, so that perceptual relations *in the blend* become conceptual relations. One example of anchoring in real life is queuing. Many cultures have developed an integration network that has people arriving at a place – for instance, to buy tickets for a concert – in one mental space, and natural numbers, or simply a sequence of slots, in another mental space. People are mapped onto numbers as they arrive, thus establishing correspondences across these mental spaces. If we project these correspondences into a blended space, we can fuse person, arrival event, and number. Now, in this newly integrated scenario, we can combine arrival order and cardinal numbers to come up with ordinal numbers or ordered slots, and we can assign those slots to people, giving rise to turns. Although it might seem trivial, the notion of turn requires a complex integration network of mappings and inferences, with emergent structure that is difficult to grasp at first. Children, for instance, do not arrive at a playground with an innate notion of turn: they need to learn it through social interaction.

But even if we have arrived at a notion of turn, we still do not have queuing. Turns may be difficult to apply if people are scattered. Now the culture, always dynamically defining goals and pursuing greater efficiency, imports additional structure into the network: a linear path with A-to-B directionality. In the blend, we can align people along this path, following their turns. If we convince those people of adopting this cultural practice, they will *enact* (Stewart et al., 2011) this material anchor for the blend and align themselves accordingly. Then we will be able to “see” the turns – who is first or second or fifth – just by looking at their place in the line they have formed. Queueing has now emerged through the anchoring of the turn blend on the linear path (Hutchins, 2005, p. 1559–1562). Material anchors for conceptual blends are everywhere. They can be both perceived and acted upon, and interaction with them is especially useful to organize complex meaning networks, from using clocks, sundials, or calendars to see the time to computer interfaces allowing one to “drag” files into folders.

Blending theory is particularly useful for providing insight into the intricacies of seemingly trivial and conventional cases of meaning construction, including complex concepts such as time. Everyday time expressions across multiple languages – including French and Greek, the major languages of Xenakis – rely on the anchoring of time by means of a mentally-simulated scene that presents a series of selected, *ad hoc* features. All objects (events) are aligned and typically move at a regular, fixed speed; all observers (time experiencers) are on the same spot. Objects can move toward observers along with the timeline, as in a conveyor belt, or be static, observers can move – if objects are static – or be static – if objects move – so that the distances on the path, covered at a regular pace, become durations, and the motion can thus be experienced from an ego-moving, object-moving, or external perspective (Fauconnier and Turner, 2008).

When we refer to any aspect of this network for time motion, we know that we are not talking about a regular physical-motion scene, but about one that has been narrowly defined and prepared to create inferences about time. Once we have this configuration in mind, cultures can create numerous expressions that point to a variety of its properties, using them to create temporal meanings. We can talk about “a long time” (e.g. French *longtemps*, although in Greek quantity is preferred to stretch: πολύ χρόνο, πολύ ώρα), about events “coming” toward observers, such as the “arrival” of spring (*le printemps est arrivé*, ήρθε η άνοιξη), about observers approaching events, as when we are getting close to being on or inside a date-location (*Nous sommes déjà en 2020*, είμαστε πια στο 2020), and about time passing (*let temps est passé*, έχει περάσει ο χρόνος), from which the nouns and adjectives referring to time as *past* or *to come* – the meaning of the Latin word *futurus*. Ample evidence from linguistics and psychology has exposed the detailed ways in which these mappings, alongside the cross-linguistic variations for activating them and the perceptual patterns to anchor them, influence human behavior in multiple time-related tasks and creative activities across cultures (Boroditsky and Ramscar, 2002; Weger and Pratt, 2008; Fuhrman and Boroditsky, 2010; Coulson and Pagán Cánovas, 2013; Pagán Cánovas et al., 2015).

Material anchors are useful for discussing agency in musical practices (Zbikowski, 2019), such as the presence of timelines in composition (Besada and Pagán Cánovas, 2020). In the forthcoming discussion of the compositional practices around *Psappha*, we analyze material anchoring in a complex conceptual integration network for time, built *ad hoc* to serve the purposes of music composition, but nonetheless based on the basic patterns that we have just outlined. Our approach will expand upon current knowledge of time and enaction in contemporary music (Kozak, 2020) from the side of composition. For this, we will be relying on visualizations from Xenakis’ sketches as well as on his theoretical writings. Before we discuss how the principles and patterns of conceptual integration make his creative process possible, we introduce sieve theory and outline Xenakis’ personal interpretation of it. Then, we analyze the application of sieves to the composition of *Psappha* through the interaction of two material anchors for complex time blends: the timeline and the gearwheel.

## From Sieve Theory to Xenakis’ Compositional Sieves

**Figure 1:**
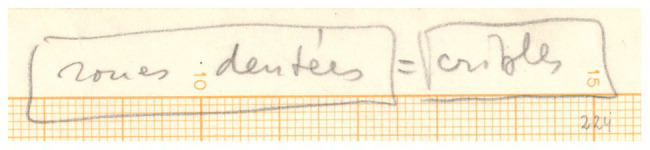
Reference to gearwheels in Xenakis’ sketches for *Psappha*. © Iannis Xenakis’ family. Reproduced by permission.

**Figure 2:**
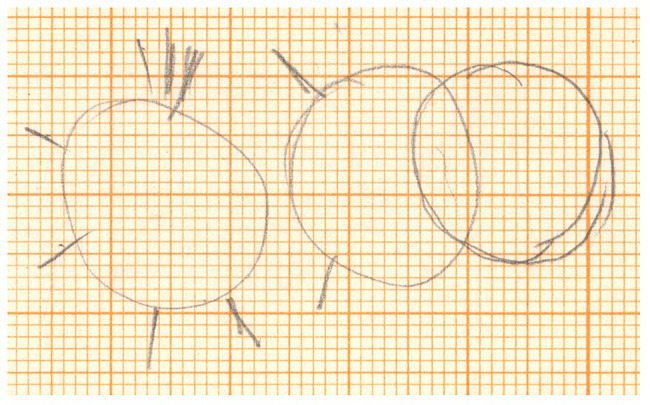
Gearwheel outlines in Xenakis’ sketches for *Psappha*. © Iannis Xenakis’ family. Reproduced by permission.

Our case study is a specific implementation of Xenakis’ sieve theory. The composer used the term “gearwheels” to refer to his adaptation of this particular mathematical method *in Psappha* (Barthel-Calvet, 2000, p. 169). Among the sketches preserved for this score, there is one in which he wrote “*roues dentées* = *cribles*” – i.e., “gearwheels = sieves” in French – (Figure 1). Another sketch shows a drawing of schematic gearwheels (Figure 2). The few specific studies concerning the sieves for *Psappha* (Flint, 1993, 2001) are based on the scrutiny of these sketches but contain no mention to the subject of gearwheels. They probably went unnoticed or were considered as anecdotal by the scholar. After all, wheel-like diagrams were mainly related to other issues he dealt with in the early 1950s, since he started to reconsider serial music techniques for his own compositional purposes (Barthel-Calvet, 2003, 2011).

The transfer from visual elements to musical ideas in Xenakis’ creative practices has been already discussed, for his architectural experiences (e.g., Barthel-Calvet, 2001, 2009; Kiourtsoglou, 2017) as well as for the computational ones (e.g., Weibel, 2020). Even a brief approach to the anchoring features of Xenakis’ compositional practices has been recently published (Besada, 2020). By contrast, we have not found detailed discussions of visualizations in the application of sieves.

### Mathematical Roots

Sieve theory is a branch of number theory that estimates the members of a subset of natural numbers or integers, sometimes also predicting the relative size of the subset, by means of iterative filtering algorithms (Halberstam and Richert, 1974; Greaves, 2001; Cojocaru and Murty, 2006). Its most famous case is the sieve of Eratosthenes for finding prime numbers (Bays and Hudson, 1977). We may summarize it in a few steps. Ignore number 1 and keep number 2, it is a prime number. Cross out all multiples of 2 – i.e., even numbers – and continue to the next natural, which is 3, again a prime number to be kept. Cross out all multiples of 3 – unless already eliminated, e.g., 6 – and continue to the next natural, 4, which had already been taken out. Then jump to the next natural, which is 5, and iterate the process *ad infinitum*.

Apparently, Xenakis’ sieve-based compositional technique would be applying strictly abstract mathematical operations to the organization of sound. He rarely provided descriptions based on visual imagery in his remarks upon sieves – although we reproduce a very eloquent example below. Just like many other ideas developed by contemporary composers, this technique may at the first seem disembodied, even disconnected from musical creativity itself, at least in the initial stages of composition. However, goal-oriented, embodied cognitive processes are at work since the inception of the composer’s imaginative process for *Psappha*. Seemingly exotic – from the cognitive point of view – examples such as Xenakis’ sieves share the basic cognitive operations of conceptual integration with any other product of higher-order human cognition. With other, less unusual instances of temporal meaning construction, such as making sense of a clock or a timeline or of conventional expressions of time-related feelings or intentions, Xenakis’ sieves also share much more specific patterns that arise when conceptual integration meets specific purposes, contexts, and practices. But before we delve into those patterns, let us examine his adaptation of sieve theory for compositional purposes and its connection with standard time concepts as well as with his own ideas about time in music.

Xenakis’ idea while developing his compositional sieves was to hold analogies between musical features – such as the distribution of pitches and beats – with the numerical intervals of complex mathematical sieves admitting a decomposition into elemental cyclic sequences, unlike the Eratosthenes method, which goes on *ad infinitum*. He developed this method since the mid-1960s and discussed it through several essays (Xenakis, 1965, 1966, 1967, 1968, 1988, 1990) without any reference to the term “gears.” One of his articles provided an important account of several compositional choices around his piece *Nomos Alpha* (1965–1966) for cello solo that helped several analysts delve into its mathematical features (Vandenbogaerde, 1968; DeLio, 1980; Vriend, 1981; Solomos, 1997; Peck, 2003). Other scholars have equally approached his sieves seeking to analyze further pieces within Xenakis’ oeuvre (e.g., Flint, 1993, 2001; Bertocchi, 2001; Pace, 2001; Squibbs, 2002, 2003; Exarchos, 2007; Gibson, 2011, p. 81–114), to understand the mathematical details of the composer’s approach (e.g., Gibson, 2001; Jones, 2001; Exarchos, 2009), to contextualize the relationships between this approach and others he already developed (e.g., Barthel-Calvet, 2012; Hoffmann, 2019), to derive computational models (e.g., Agon et al., 2004; Ariza, 2005), to borrow sieves for their own compositional purposes (e.g., Tipei, 1987, 1989), or as a formal framework for analyzing other repertoires (Noll et al., 2006). To the best of our knowledge, there is only one work providing cognitive remarks upon Xenakis’ sieves, and again in quite general terms (Besada, 2019, p. 265–267).

### Compositional Bases

Xenakis used sieves for several creative purposes. His first article on this topic sought to provide a universal method for describing the pitch structure of any scale (Xenakis, 1965). Subsequent articles on sieves equally focused on pitches, but he also remarked that the formalization might serve to organize other features of sound (Xenakis, 1966, 1967, 1968). His last two essays on sieves put the emphasis on time (Xenakis, 1988) and on their rhythmic exploitation (Xenakis, 1990). The former articles are the most relevant for understanding the axiomatic background of Xenakis’ perspective. His starting formal step was to adapt Peano axioms – i.e., the logical foundations for the arithmetic of natural numbers (Segre, 1994) – within a musical context:

*“Preliminary terms*. *O* = the stop at the origin; *n* = a stop; *n'* = a stop resulting from elementary displacement of *n*; *D* = the set of values of the particular sound characteristic (pitch, density, intensity, instant, speed, disorder…). These values are identical with the stops of the displacements.
*First propositions (axioms)*.

1. Stop *O* is an element of *D*;
2. If stop *n* is an element of *D* then the new stop *n'* is an element of *D*;
3. If stop *n* and *m* are elements of *D* then the new stop *n'* and *m'* are identical if, and only if, stops *n* and *m* are identical;
4. If stop *n* is an element of *D*, it will be different from stop *O* at the origin;
5. If elements belonging to *D* have a special property *P*, such that stop *O* also has it, and if, for every element *n* of *D* having this property the element *n'* has it also, all the elements of *D* will have the property *P*” (Xenakis, 1992, p. 194).

Already at this early stage, we find a dynamic, goal-oriented reuse of the mathematical notions for establishing musical concepts, which necessarily rely on embodied or sensorimotor processes for their structure. Indeed, for the case of pitch, this reinterpretation of Peano axioms depends on schemas resulting from basic spatial cognition such as scalar structures or motion along a path (Besada, 2019, p. 266), on which arithmetic is generally grounded (Lakoff and Nuñez, 2000, p. 68–74). This grounding is eloquently formulated through mental imagery engaging with a material anchor in one of Xenakis’ essays. He mentally visualized the organization of pitches following the total order of natural numbers as “a strip of paper with equidistant holes, which when placed over a special piano keyboard will locate keys separated by any elementary displacement” (Xenakis, 1966, p. 49). So far, the spatial cognition schemas applied to these conceptualizations were used to organize relations between sounds without introducing time.

When the sieve technique was used for rhythmic organization, the mental pattern of the timeline as a material anchor for temporal meanings arose. We can see it in another text by Xenakis on sieves, where he used the major standard mappings that shape the blended scenario of spatialized time recurrent across conventional conceptualizations: motion along the path maps onto time itself, which can now pass or flow; dots on the timeline path map onto events, which become landmarks that can thus be visualized in a sequence, a basic function of the timeline. As the composer said, “[t]hanks to separability, […] events can be assimilated to *landmark points* in the flux of time” (Xenakis, 1992, p. 264). In one of his earliest articles, he had already mentioned, quite explicitly, another standard mapping for the spatialized-time blend: the correspondence between distances on the line and durations. This blend gives rise to expressions such as “long/short time,” to the segmentation of timelines into periods, to proportional divisions in linear or circular calendric representations, and so forth. In this text – first published in German (Xenakis, 1956) and thus prior to the composer’s conception of musical sieves – time is “a straight line on which the points corresponding to the variations of other components [of a phenomenon] are marked”; consequently “[t]he interval between two points is identical with the duration” (Xenakis, 1992, p. 12).

Right from the start, Xenakis’ ideas about the organization of time in his musical compositions needed to rely on the timeline and other standard conceptions of spatialized time, all of them grounded in embodied cognition and made possible by conceptual integration, including processes of extended cognition for anchoring thought on perceptual information. Awareness of these templates for conceptual integration is crucial for understanding how any individual operates on them to serve specific purposes, and in this case, how Xenakis exploited their possibilities and explored their boundaries, in his effort to create innovative time effects of aesthetic value. Examining such innovative practices provides crucial insights precisely on the boundaries being pushed, on the general nature of these representations, and on the cognitive operations underlying creativity and meaning construction in general. Within this “timeline context,” it is now useful to examine how Xenakis moved forward in his technique of sieves, in a goal-oriented process that is partially structured by the mental timeline and the drive toward achieving musical effects, through the analogical arrangement of events as landmarks on a number line.

Xenakis’ reinterpretation of Peano axioms is followed in his essays by a description of methods for obtaining families of subsets of numbers and for operating with them. The elemental subsets are built *via* what mathematicians define as congruences of modular arithmetic (Jones, 1964). As the composer explained, “[t]wo integers *x* and *n* are said to be congruent modulo *m* when *m* is a factor of *x* − *n*” (Xenakis 1992, p. 195). For instance, 13 and 1 are congruent modulo 12 because 13–1 equals 12. Modular relationships induce classes of equivalence – i.e., subsets of related elements – on natural numbers. For instance, the classes of natural numbers congruent with 1 modulo 12, and with 5 modulo 8 are:

$$12_{1}=\left\{1,13,25,37,49,61,\ldots \right\}$$

$$8_{5}=\left\{5,13,21,29,37,45,\ldots \right\}$$

We are preserving one of Xenakis’ most common notations, wherein the large number stands for the modular constant, and the sub-index for the smaller representative of the class – the residue. Each residual class of equivalence incorporates a sub-periodicity – a particular kind of sieve – within the progression of naturals.

Xenakis’ next step was to carry out basic Boolean operations on the elemental sets, mainly *union* – the gathering of elements – and *intersection* – the filter of common elements. In addition, he also took into account the *complementary set*, i.e. the one with the lacking naturals within the starting set. These formal operations reflect container image schemas and have therefore an embodied origin (Lakoff and Nuñez, 2000, p. 121–131). Therefore, we have not actually been dealing with disembodied processes at any point of this creative development. Considering the previous examples, their respective union and intersection are:

$$12_{1}\cup 8_{5}=\left\{1,5,13,21,25,29,37,45,49,\ldots \right\}$$

$$12_{1}\cap 8_{5}=\left\{13,37,61,\ldots \right\}$$

Finally, Xenakis added further transformations beyond basic logics of mathematical set theory, which he coined as *metabolae*. His main metabolae entailed the modification of the modular numbers or indices. For instance, 13_1_ would be a metabola of 12_1_, just replacing number 12 by 13 but leaving sub-index 1 invariant.

## Integration and Anchoring in the Sieve Technique For Xenakis’ *Psappha*

Let us pursue for a moment a strictly formal analysis of this compositional technique, without taking cognitive processes into consideration. Among Xenakis’ sketches for *Psappha*, one of them reproduces the complex calculations for the sieve – henceforth *S* – that he used for distributing the beats during the 40 first pulses of his score. Flint (1993, p. 232) transcribed Xenakis’ formulae as follows:

$$\begin{array}{l} S=\left[\left(8_{0}\cup 8_{1}\cup 8_{7}\right)\cap \left(5_{1}\cup 5_{3}\right)\right]\cup \;\left[\left(8_{0}\cup 8_{1}\cup 8_{2}\right)\cap 5_{0}\right]\cup \\ \;\cup \left[8_{3}\cap \left(5_{0}\cup 5_{1}\cup 5_{2}\cup 5_{3}\cup 5_{4}\right)\right]\cup \;\\ \;\cup \left[8_{4}\cap \left(5_{0}\cup 5_{1}\cup 5_{2}\cup 5_{3}\cup 5_{4}\right)\right]\cup \\ \;\cup \left[\left(8_{5}\cup 8_{6}\right)\cap \left(5_{2}\cup 5_{3}\cup 5_{4}\right)\right]\cup \left[8_{1}\cap 5_{2}\right]\cup \left[8_{6}\cap 5_{1}\right] \end{array}$$

The expression above is extremely complex. Flint proceeded by applying the distributive property – i.e., by ungrouping the expression for obtaining its elementary constituents. It leads to a large list of 27 intersections of two sieves each. As 8 and 5 are coprimes – i.e., they do not share a common prime factor – there is only one solution for each sieve from 0 to 39. Finally, she gathered all the elemental solutions in a single set (Flint, 1993, p. 232; we add number 22, which she forgot to include in the list):

$$S=\left\{\begin{array}{l} 0,1,3,4,6,8,10,11,12,13,14,16,17,19,20,\\ 22,23,25,27,28,29,31,33,35,36,37,38 \end{array}\right\}$$

If we stick to this type of analysis, the insight gained into the compositional process is limited. We already showed that Xenakis had to rely on standard, everyday integration templates for forming time concepts and for anchoring them on perceptual structures in order to interact with them productively. Without the timeline structure, and without the aesthetic motivation to distribute events along a timeline following certain patterns, we would have never had any sieve technique to apply to the composition of *Psappha* in the first place. Now, in order to understand how Xenakis got to his final compositional choices, we need to take into account his explicit reference to gearwheels. This second material anchor provides the sieves with a material structure that turns them into an actionable object. The resulting mental space, in which gearwheel-sieve and timeline can now interact, gives rise to a rhythmic structure that would have been unavailable from either timeline or sieves separately.

### Sieves, Gearwheels, and the Elemental Cycles

As we said, Flint’s analysis disregarded the textual and visual references to gearwheels in Xenakis’ sketches. However, if we bring in the “materialization” of sieves as gearwheels, new light can be shed on Xenakis’ creative process. For one thing, the material anchoring of the sieves, and the manipulations it affords, becomes a central part of the analysis. The choice of gearwheels to anchor sieves is well motivated in cognitive terms, since it is grounded on a set of generic properties shared by both sieves and gearwheels. Material anchors are not arbitrary symbols but objects or spatial configurations that arise from interactions with the world that have been found to facilitate the cognitive process. Just like cultural evolution leads to solutions such as the timeline or the queue, whose affordances serve chronology and sequence arrangement, Xenakis got to the gearwheel because of its potential for matching the cyclic sequences that he was pursuing with the sieves.

Circular templates for representing musical rhythm over time are found in essays of ethnomusicology (e.g., Becker, 1980; Anku, 2000), music theory (e.g., Toussaint, 2005, 2013; Benadon, 2007), and the psychology of music (London, 2004, p. 64–69). These representations are consistent with the ubiquity of circular or spiral schemas that cultures use to anchor natural cycles (Overton, 1994; Yamada and Kato, 2006; Laeng and Hofseth, 2019). All these anchors cohere with the blending template that allows human to compress regular sequences of events into cycles, giving rise to our cyclic notions of day, year, and so forth (Fauconnier and Turner, 2002, p. 195–198). All these representations are adjusted to the main goal of dividing the continuous flow of time – within or without music – into discrete units with which it is possible to operate. Xenakis goes one step further here. By creating a complex interaction of the gearwheel and the timeline anchors, he produced a combination of cyclic and linear time that gave rise to the particular rhythmic structures in *Psappha*.

Let us now examine how Xenakis’ cyclic model works. Modular arithmetic is generally introduced to young students as the “clock arithmetic.” Consider the sieve 12_1_ provided above and think of a clock sphere. The first two elements of this sieve are 1 and 13; we read both values in the same position on the clock, for 1AM and for 1PM. Applying analogous protocols, Xenakis’ elemental sieves for *S* can be equally projected onto a circular template akin to the clock shape, but modifying the number of its equal divisions. Consider for instance the elemental sieves modulo 5 in *S*. They can be anchored onto a circumference split into five equal arcs, starting with a point in a position equivalent to noon-midnight in a clock. This point may receive the label 0, and the process of labelling with values 1, 2, 3, and 4 is performed clockwise. With this support, any elemental sieve modulo 5 is amenable to a material representation. For instance, the sieve 5_0_ is represented by a mark on point 0. Similarly, the union of elemental sieves sharing the same modulo is easily combined within the same support. For instance, the union of elemental sieves $\left(5_{2}\cup 5_{3}\cup 5_{4}\right)$, which is found in the previous formula, is represented by marks on the points 2, 3, and 4.

It is easy to visualize a clock hand – for instance marking seconds – cyclically moving through the aforementioned sieve and union of elemental sieves. The person mentally performing this action may also have an internal feeling of beats when the clock hand reaches a marked point. It would be a beat every 5 s for the sieve 5_0_, and cycles of three consecutive beats followed by two unbeaten pulses for the union $\left(5_{2}\cup 5_{3}\cup 5_{4}\right)$. As the notion of “clock arithmetic” is quite common, Xenakis was probably aware of it; he opted however for comparing his compositional process with gearwheels. These devices are a substantial part of mechanical clocks and their rotational motion image is widespread in Western culture. Unlike the usual shape of clocks, which is often a smooth, unaltered circumference, gears have teeth. Gear teeth exist for interaction with other devices or surfaces and may leave marks. This is where the motivation to interact with the timeline kicks in.

**Figure 3:**
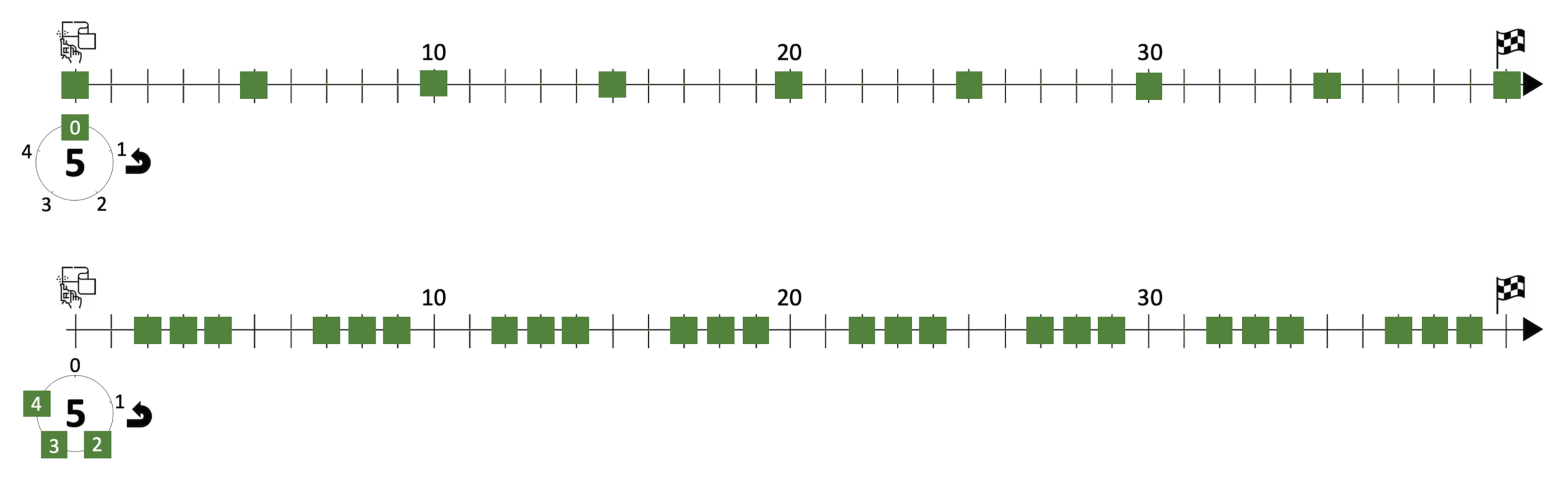
Schematic representation of the scrolling motion of elemental sieves.

In the novel mental space resulting from the integration of these two already complex blends, the affordances of the gearwheel-sieve meet those of the musical timeline. Within the context of Western musical notation and its horizontal timeline, where figures are annotated as landmarks on a two-dimensional surface, it is not difficult to mentally imagine a gearwheel freely rolling on such a surface and leaving a line of marks or fixedly rotating on itself to imprint the marks on a scroll sheet unfolding a stave or any other akin representation. We have visualized this idea in Figure 3: the upper circle stands for the elemental sieve 5_0_, in which the mark on point 0 is represented by a square, for transforming the previous clock-like template into a gear-like one. We can imagine the marked circle rolling counterclockwise under the timeline and dropping a landmark point – “on the flux of time,” as Xenakis could have said – when the point of the sieve cyclically meets the straight timeline. We have unfolded the process eight times for matching with the first 40 pulses of *Psappha*. The diagram below in the same figure is an equivalent representation for the union $\left(5_{2}\cup 5_{3}\cup 5_{4}\right)$. For these kinds of “scrolling motion” mental images, the gear-like shape proves more suitable than that of the clock.

### The Gearwheel-Timeline System in Xenakis’ Sieve-Based Composition

**Figure 4:**
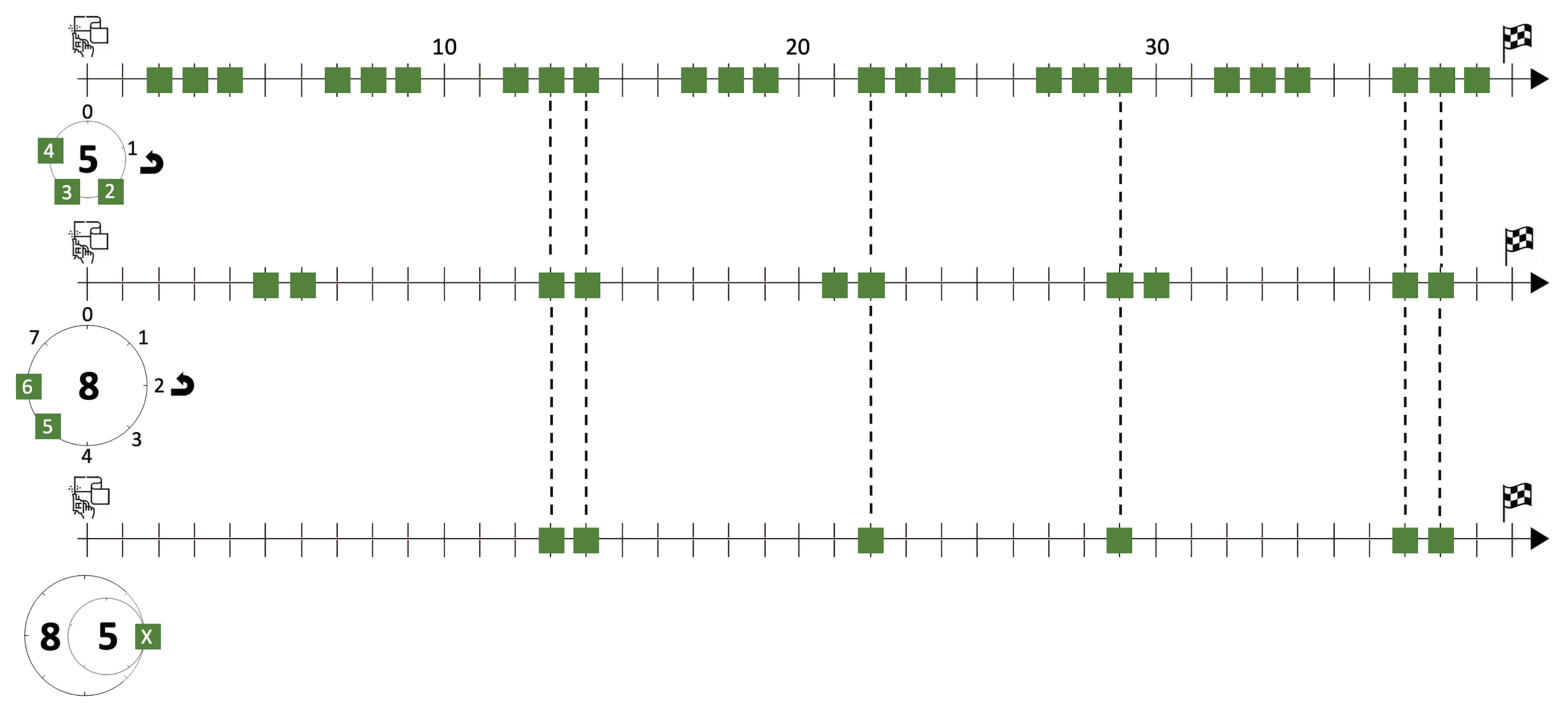
Schematic representation of the scrolling motion of elemental sieves and their intersection.

Now let us examine whether this idea of enaction through imaginary material anchors can illuminate some of Xenakis’ choices during his compositional process. In Figure 3, we have just dealt with sieves made of elemental components sharing a modulo. In the mathematical expression for *S*, all these unions are expressed within parentheses. Additionally, *S* also incorporates elemental sieves with modulo 8. Elemental sieves or blocks of sieves sharing modulo 5 are confronted with similar objects sharing modulo 8 *via* intersection in an upper level – bounded within brackets. It is the case, for example, of the subexpression $\left[\left(8_{5}\cup 8_{6}\right)\cap \left(5_{2}\cup 5_{3}\cup 5_{4}\right)\right]$, which is represented in Figure 4. Its upper diagram simply reproduces the lower diagram of Figure 3. Below, a similar configuration is provided for the union $\left(8_{5}\cup 8_{6}\right)$. The unfolding marks over parallel timelines can be used for estimating the intersection, just by checking the aligned landmark points dropped on both straight lines.

We have reproduced the result of the intersection in the lower diagram of Figure 4. Instead of accompanying its timeline with its implicit forty-teeth gear-like template, we have represented it with a diagram, which stands for the interaction of the gears above. The intersection can still be obtained through extremely complex operations based on Boolean algebra, as shown above, but Xenakis’ mental image of gearwheels, which made its way to one his sketches, provides a plausible insight into how the creative conceptualization may have been imagined. A dynamic interaction with familiar material anchors is just as possible virtually as in a scenario of direct perception. Whether Xenakis only used mental imagery or also enacted the gearwheel-timeline interaction in further drawings unavailable to us, we cannot know, but it is indeed cognitively plausible to imagine both gears rolling in parallel across the same timeline. In this situation, they would only drop a landmark point onto the straight line when both gears are simultaneously activated for doing so.

**Figure 5:**
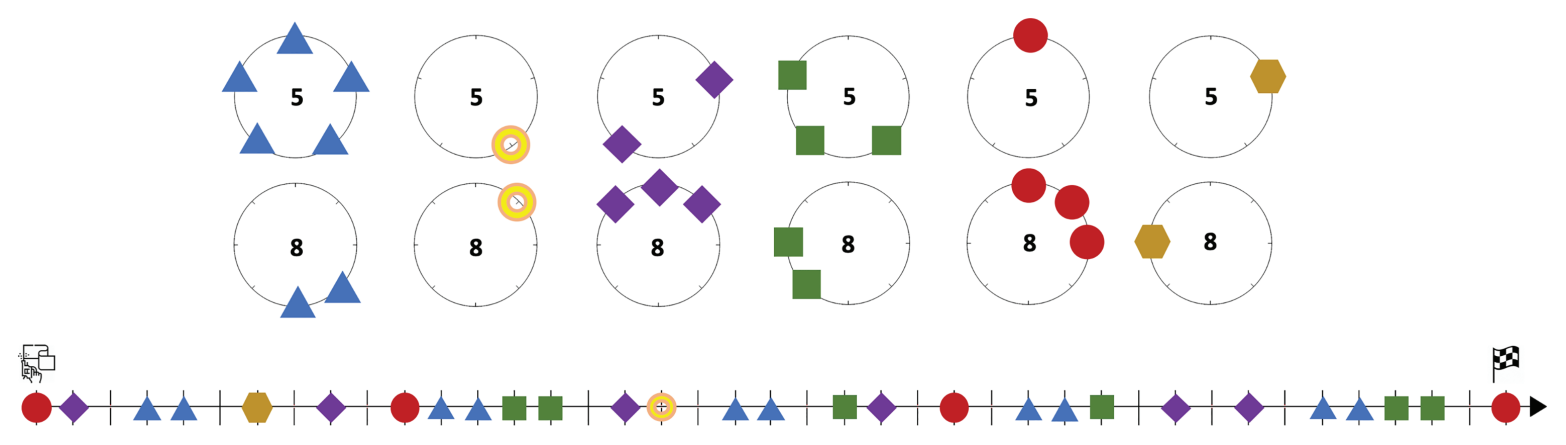
Gearwheel representation of the elemental sieves leading to *S* and their unfolding onto a timeline.

We are ready for jumping to the uppermost level of Xenakis’ sieve. Again, it is in principle possible to obtain the same results through complex operations based on Boolean algebra, but now the exclusive use of that option is becoming increasingly unrealistic, because it requires a much stronger cognitive workload than the gearwheel alternative. It would be similar to a particular case of ship navigation, not assisted by computational devices, where humans would choose to calculate relative positions, bearings, and routes exclusively through arithmetic operations, without resorting to the perceptual location of landmarks and the customary multimodal interaction with charts, scales, triangles, and so forth (Hutchins, 2011). The enactive solution, whether Xenakis actually made drawings or ran it through mental simulation, is not only much more cognitively plausible but also more realistic when it comes to doing creative work with time concepts, since it allows for a dynamic interaction with one’s own ideas about temporality and rhythm as the creative process unfolds. Therefore, in this step, the union entails the simultaneity of several pairs of gearwheels – as those we already provided in Image 4 – conforming a kind of “complex clockwork” to be confronted with the timeline. Every pair of gears drops its landmark points, and the assembly of them all completes the action of the sieve. The formula provided above, in which there are seven bracketed subexpressions, admits the merge of two of them by means of the distributive property. In doing so, *S* is reduced to six subexpressions, which are depicted in Figure 5, with the following color-shape code for both the teeth of the gears and the landmark points on the timeline:

- Blue triangles: $\left[\left(8_{3}\cup 8_{4}\right)\cap \left(5_{0}\cup 5_{1}\cup 5_{2}\cup 5_{3}\cup 5_{4}\right)\right]$.
- Yellow rings: $\left[8_{1}\cap 5_{2}\right]$.
- Purple diamonds: $\left[\left(8_{0}\cup 8_{1}\cup 8_{7}\right)\cap \left(5_{1}\cup 5_{3}\right)\right]$.
- Green squares: $\left[\left(8_{5}\cup 8_{6}\right)\cap \left(5_{2}\cup 5_{3}\cup 5_{4}\right)\right]$.
- Red circles: $\left[\left(8_{0}\cup 8_{1}\cup 8_{2}\right)\cap 5_{0}\right]$.
- Ocher hexagons: $\left[8_{6}\cap 5_{1}\right]$.

Enaction through gearwheels is by no means incompatible with the use of modular arithmetic, which is necessary for calculating the accurate elemental values of the sieve step by step. Indeed, Flint’s paper summarizes the calculations found in Xenakis’ preserved sketches. But, as we are showing, exclusive use of this non-enactive procedure becomes more implausible as we delve into the intricacies of the compositional process. This hypothesis receives more support when we look into the “beat-zipping effect” in the opening of *Psappha*.

### Gearwheels as Zippers

**Figure 6:**
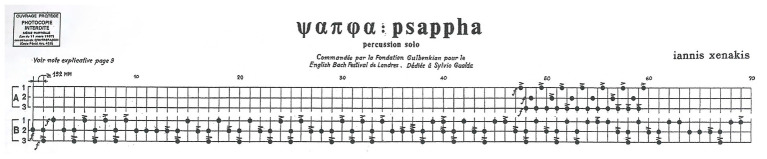
*Psappha* (first 70 pulses) by Iannis Xenakis. © 1976 Éditions Salabert. Reproduced by kind permission of Universal Music Publishing France and Éditions Salabert.

Xenakis’ score for *Psappha* is not written within the current standards of Western music notation. The composer gave Sylvio Gualda – the percussionist Xenakis wrote the piece for – a fair copy with vertical segments like note stems, akin to the notations on graph paper of his sketches, but Gualda found this document too hard to read (Lalitte, 2018, 19′01″–19′40″). Consequently, at Gualda’s request, Xenakis had to opt for a new notational protocol, which is in addition more helpful, visually speaking, for our cognitive discussion. Each system of the published score – as the first one shown in Figure 6 – is almost a grid in which circled marks akin to noteheads are distributed. In this passage, each horizontal line, namely A1, A2, A3, B1, B2, and B3, is assigned to a wooden or membranophone instrument. These lines should be regarded as simultaneous timelines, just like those in our previous figures. They resemble the time-unit box systems developed by ethnomusicologists (Koetting, 1970). In addition, Xenakis explicitly anotated that the minimal segment of timelines stands for the metronomic pulse 152 MM or faster. The displayed noteheads therefore represent the landmark points Xenakis assigned for each beat that the percussionist has to play. Other symbols in the score for dynamics and articulation are more conventional.

We may henceforth ignore the lines for instruments A1, A2, and A3 as they do not come into the scene during the first 40 pulses and are not an outcome of any sieve. Conversely, the music during this lapse of time for instruments B1, B2, and B3 strictly depends on sieve *S*. Just compare the straight timeline in Figure 5 and the noteheads Xenakis wrote for instrument B2 from pulse 0 to pulse 39. They are exactly in the same place, which means that the outcome of the sieve has been directly transcribed into the line for this instrument. The distribution of noteheads for instruments B1 and B3 during this passage is a consequence of that first choice. On the one hand, compare the lines for instruments B1 and B2: there are noteheads for instrument B1 exactly in the pulses, which are not beaten by instrument B2. On the other hand, compare the lines for instruments B1 and B3: the lower layer replicates the beat pattern of the upper one, but preceding it by one pulse.

Instrument B3 has of course an impact on the aural appearance of the passage, but we will now focus on the relationship between instruments B1 and B2. From the perspective of Boolean algebra, it is possible to describe the elements matching with the noteheads for instrument B1 as the complementary set of the sieve *S*, that is, all the elements of a universal set – here pulses from 0 to 39 – which do not belong to the sieve. From the perspective of the gear-like templates, Xenakis’ choice can be regarded as two meshing gearwheels. It is indeed one of the main reasons justifying the shape of these devices: when two gearwheels mesh, their respective teeth are counterbalanced, i.e., each tooth of a gear occupies the empty space between two contiguous teeth of the confronted gear. Consider the mental image of linearly unfolding these gear-like shapes: they would fasten like zippers.

It is true that complementarity in Boolean terms played an important role in the conception of Xenakis’ early pieces (Xenakis, 1963, p. 200–208; Squibbs, 2000; Wannamaker, 2001). As stated in a previous section of our analysis, Xenakis also mentioned complementarity in his theoretical essays as a tool for calculating sieves, but rather as a secondary feature of Boolean algebra. Indeed, the union and the intersection were ubiquitous operations within Xenakis’ sieves; conversely, complementary sets were not always present. Among the cases in which complementarity was summoned in this compositional context, *Psappha* stands as the most eloquent one, aurally speaking, because of its straightforward implementation.

This effortless approach, mathematically speaking, to the complementary set of a complex sieve is precisely found in the piece that Xenakis expressly related to gearwheels in its compositional sketches. We surmise therefore that the gear-like template was crucial for conceiving a kind of “beat zipping” between instruments B1 and B2 in the opening of *Psappha*. Once more, enaction through material anchors provides a plausible cognitive account of how Xenakis’ thought may have led to this choice. The use of modular arithmetic would have been restricted to obtaining accurate calculations where necessary, rather than to reach the beat-zipping insight, which is so readily available from the enactive process.

### Metabolae and Prosodic Rhythm

Xenakis also transformed his sieves by means of numerical transpositions or the metabolae technique. The latter was also exploited for *Psappha*. Let us look back to the score excerpt we provided in Figure 6. We keep ignoring instruments A1, A2, and A3. As the cycle of the complex sieve finished in beat 39, it could have started again at beat 40. If we compare the music of the very opening beats and what Xenakis wrote from beat 40, it is quite akin; however, after a few strokes, both patterns strongly diverge. This happens because the distribution of noteheads for instruments B1, B2, and B3 currently depends on a metabola of *S*. Xenakis operated a change of the moduli – 8 becomes 7 while 5 becomes 6 – and incorporated a few adaptations for having elemental cycles akin to the former sieve. The formula of his new sieve *S*′ is (Flint, 1993, p. 232):

$$\begin{array}{l} S\prime =\left[\left(7_{0}\cup 7_{1}\cup 7_{7}\right)\cap \left(6_{1}\cup 6_{3}\right)\right]\cup \;\left[\left(7_{0}\cup 7_{1}\cup 7_{2}\right)\cap 6_{0}\right]\cup \\ \;\cup \left[7_{3}\cap \left(6_{0}\cup 6_{1}\cup 6_{2}\cup 6_{3}\cup 6_{4}\cup 6_{5}\right)\right]\cup \\ \;\cup \left[7_{4}\cap \left(6_{0}\cup 6_{1}\cup 6_{2}\cup 6_{3}\cup 6_{4}\cup 6_{5}\right)\right]\cup \\ \;\cup \left[7_{5}\cap \left(6_{2}\cup 6_{3}\cup 6_{4}\right)\right]\cup \left[7_{1}\cap 6_{2}\right]\cup \left[7_{6}\cap 6_{1}\right] \end{array}$$

**Figure 7:**
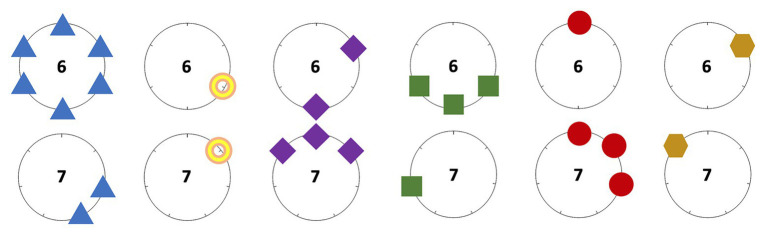
Gearwheel representation of the elemental sieves leading to *S'*.

As we did before, the expression can be subdivided into six more elemental ones. Figure 7 provides its visual interpretation through gearwheels. Its resemblance with Figure 5 deserves some discussion. Metabolae are intrinsically algebraic constructs but, in this context, they can be interpreted through the visual templates. The metabolae operated by Xenakis basically match with respectively adding or subtracting one teeth from the gear in each coupled system, for finally merging them all.

We continue ignoring instruments A1, A2, and A3. Although the passage from beat 0 to 39 and the one from 40 to 79 are quite different in terms of distribution of noteheads, the listener may find strong resemblances, aurally speaking. Of course, the limitation of pulsed beats to only three instruments is the main reason for this, but a closer look to the score allows us to also detect structural parallels. Just consider the first three pulses: the initial one is beaten by instrument B2, the second one is simultaneously beaten by instruments B2 and B3, and the last one is beaten only by instrument B1. This rhythmic cell in pulses 0–2, which may be perceived as an iambic pattern, is repeated 14 more times within the passage in Figure 6: in pulses 3–5, 13–15, 16–18, and so on.

**Figure 8:**
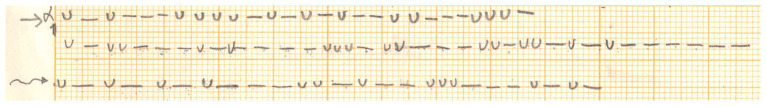
Metric feet in Xenakis’ sketches for *Psappha*, also printed in Barthel-Calvet (2000, p. 255 of the appendix). © Iannis Xenakis’ family. Reproduced by permission.

The choice of the Greek metrical foot for defining this pattern is not our suggestion. Surely informed by Xenakis’ sketches (Figure 8), Flint’s analysis mentions “iambic-based schemes” and provides a table of their distribution with the conventional ∪__ notation for iambs (Flint, 1993, p. 229). Psappha is the Aeolic name of Sappho: Xenakis’s title honors the famous Greek poet. However, the rhythmic patterns in *Psappha* are not imitating the hendecasyllabic verses of a Sapphic stanza (Mâche cited in Barthel-Calvet, 2000, p. 317).

Although Flint discussed the formal aspects beyond the emergence of iambic feet, she did not really problematize this issue. First, Xenakis’ sieves were chiefly aimed at this purpose. If we count the elements of *S*, there are 27 numbers, which is about two-thirds of the whole cycle of 40 beats. A distribution of two-thirds of beats for instrument B2 – along with the mechanism for obtaining those for instruments B1 and B3 – is the most suitable quantity for obtaining a large number of iambic patterns. A smaller or a bigger size would entail, statistically speaking, less iambs. Second, the effect of the metabola discussed above and other methods – beyond the sieve technique (Flint, 1993, p. 230) – make iambic feet spread in an unpredictable way. *Psappha*’s combination of recurrence and unpredictability, with great frequency of iambs, allows for an evocation of the flow of tonic and non-tonic syllables in speech, especially in the prosody of Indo-European languages, including French and Greek. It also perhaps resembles the sequences of long and short syllables in ancient Greek, with or without a metrical pattern.

What Xenakis’ precise goal was, that we do not know, but the existence of a purpose in the manipulation of the sieves seems evident. Once more, we see that the engagement with the material anchors is purposeful, driven by the search of a particular musical effect rather than mechanically applying abstract formulations. This goal-orientedness is a central characteristic of any blending or anchoring process. Another defining feature of conceptual integration, which we can see here quite well, is dynamicity: goals and conceptual operations mutually modify one another as the creative process unfolds. This “emergent design” allows the composer to opportunistically seize the new possibilities discovered through interactive engagement with others, with objects in the environment, or with virtual structures that present interactive affordances, such as material anchors. Xenakis’ metabolae technique for adjusting his sieves is thus reflecting the opportunism of conceptual integration that we can witness in so many human activities, and which allows insights to arise across musical performance, face-to-face communication, and so many other examples of joint action, in complex collective dynamics such as the company culture or the film set, or in the individual acts of creation that are typical in music composition, literature, or the plastic arts.

## Conclusion

The understanding of general cognitive processes proves crucial for explaining the sophisticated, *ad hoc*, seemingly opaque compositional choices made by Xenakis in *Psappha*. To expose the connections between Xenakis’ manipulation of sieves as gearwheels and conventional time representations, we have used the frameworks of blending theory and enaction, and the notions of network integration and material anchoring. Quite surely, alternative views could be proposed, and we are also confident that future developments will result in improved theories providing better accounts of the intricacies of human creativity and imagination. But what cannot be doubted is that an understanding of the general cognitive abilities underlying all meaning construction processes is necessary to reach any fruitful insights into creative practices in any particular domain, in this case music.

This also includes an understanding of the generic purposes that guide cognitive operations, which are always dynamic and goal-oriented. In this case, it is crucial to understand the overarching goal of any integration of material and conceptual structures, which is always to compress the manifold, scattered information dispersed across the mental network – multiple time-space mappings, the standard properties of the timeline, the complex operations of sieves, and so forth – into a scene at human scale, where interaction and manipulation can produce conceptual outcomes in a straightforward way – actioning gearwheels to obtain rhythmic patterns. In this sense, Xenakis’ sieve-wheels are driven by the same forces that give rise to the clock, the timeline, or the practice of queuing. To analyze these processes, visualizations acquire great value as data. The detailed knowledge about a composer’s ideas and theoretical proposals needs to be combined with the close examination of sketches and other ethnomusicological procedures, such as the study of gesture in recorded interviews. We need to integrate all these methods to gain insight into the domain of compositional practices, which is perhaps the hardest to tackle in the field of musical creativity.

Of no less importance, we must also flip the coin and look at the other side of the methodological argument. The detailed cognitive analysis of compositional practices, especially if they are as intricate and non-typical as the ones displayed by Xenakis in our case study, is necessary not only to understand musical creativity or even creativity in general but also the fundamentals of cognition. By submitting the timeline and the standard mappings for spatialized time to considerable manipulation, in order to serve his aesthetic purposes, the Xenakis case study is also exposing the structure of these patterns, their possibilities, and their limits, in particular, how far the simulated interaction among material anchors can be taken while still retaining their major representational properties. Research on distributed cognition has indeed pointed at how a material or perceptual structure, such as a map or a computer interface, can help us to download cognitive effort, flagging this as a proof that cognition is distributed and situated, and therefore emerging from both the mind-brain-body and the interaction with the object and the environment. But we know little about the mental manipulations of these anchors, which may also take place without direct action or in the absence of the perceptual stimulus. We also need to know much more about the possibility of combining more than one anchor into a novel, more complex anchoring system, just like Xenakis did for *Psappha*.

Very much in the interdisciplinary spirit of Xenakis, who so often transferred ideas across music, architecture, mathematics, engineering, or computing, we can now go to other intellectual domains for comparison. This will allow us to shed light on both the general cognitive abilities and the specificities of musical creativity. If we create sufficient common ground, a “shared conceptual space,” where musicology can engage with the general quest for human meaning-making in the cognitive sciences, we can expect the emergent design to render exciting results about both music and cognition, just like Xenakis’ gearwheel-sieve interacts with the general timeline shared by all, giving us the unique rhythmical experience of *Psappha*.

## Ethics Statement

Written informed consent was obtained from the individual(s) for the publication of any potentially identifiable images or data included in this article.

## Author Contributions

All authors listed have made a substantial, direct and intellectual contribution to the work, and approved it for publication. A-SB-C from the perspective of musicology and music theory, CP from the perspective of cognitive science, and JB from both sides.

### Conflict of Interest

The authors declare that the research was conducted in the absence of any commercial or financial relationships that could be construed as a potential conflict of interest.
